# Delivery of Berberine Using Chitosan/Fucoidan-Taurine Conjugate Nanoparticles for Treatment of Defective Intestinal Epithelial Tight Junction Barrier

**DOI:** 10.3390/md12115677

**Published:** 2014-11-24

**Authors:** Shao-Jung Wu, Trong-Ming Don, Cheng-Wei Lin, Fwu-Long Mi

**Affiliations:** 1 Department of Chemical Engineering, Ming Chi University of Technology, New Taipei City 243, Taiwan; E-Mail: sjwu@mail.mcut.edu.tw; 2 Department of Chemical and Materials Engineering, Tamkang University, New Taipei City 251, Taiwan; E-Mail: tmdon@mail.tku.edu.tw; 3 Department of Biochemistry and Molecular Cell Biology, School of medicine, Taipei Medical University, Taipei City 110, Taiwan; E-Mail: cwlin@tmu.edu.tw; 4 Graduate Institute of Medical Sciences, College of Medicine, Taipei Medical University, Taipei City 110, Taiwan

**Keywords:** chitosan, fucoidan, nanoparticles, drug delivery, tight junction

## Abstract

Bacterial-derived lipopolysaccharides (LPS) can cause defective intestinal barrier function and play an important role in the development of inflammatory bowel disease. In this study, a nanocarrier based on chitosan and fucoidan was developed for oral delivery of berberine (Ber). A sulfonated fucoidan, fucoidan-taurine (FD-Tau) conjugate, was synthesized and characterized by Fourier transform infrared (FTIR) spectroscopy. The FD-Tau conjugate was self-assembled with berberine and chitosan (CS) to form Ber-loaded CS/FD-Tau complex nanoparticles with high drug loading efficiency. Berberine release from the nanoparticles had fast release in simulated intestinal fluid (SIF, pH 7.4), while the release was slow in simulated gastric fluid (SGF, pH 2.0). The effect of the berberine-loaded nanoparticles in protecting intestinal tight-junction barrier function against nitric oxide and inflammatory cytokines released from LPS-stimulated macrophage was evaluated by determining the transepithelial electrical resistance (TEER) and paracellular permeability of a model macromolecule fluorescein isothiocyanate-dextran (FITC-dextran) in a Caco-2 cells/RAW264.7 cells co-culture system. Inhibition of redistribution of tight junction ZO-1 protein by the nanoparticles was visualized using confocal laser scanning microscopy (CLSM). The results suggest that the nanoparticles may be useful for local delivery of berberine to ameliorate LPS-induced intestinal epithelia tight junction disruption, and that the released berberine can restore barrier function in inflammatory and injured intestinal epithelial.

## 1. Introduction

Intestinal epithelial tight junctions provide the barrier function in preventing the invasion of bacterial endotoxin and subsequent contact with the immune system. Bacterial-derived lipopolysaccharides (LPS) can cause defective intestinal barrier function which increases the risk of development of inflammatory bowel disease [1]. Berberine (Ber) is an isoquinoline alkaloid in the *Berberis* species which has many antimicrobial activities against fungal, bacterial and viral infections [2]. Berberine also exhibited potential anti-inflammatory activity both *in vitro* and *in vivo* [3]. Several studies reported that berberine promoted tightness of the intestinal epithelial tight junction (TJ) barrier and ameliorated TJ barrier impairment by suppressing the production of proinflammatory cytokines [4,5,6,7,8]. However, its application in oral administration is limited mainly due to the low local concentration, short residence time, and poor absorption in the intestinal tract [9]. To overcome these problems, the development of a drug delivery system with both mucoadhesive and pH-sensitive properties is required to increase the local berberin concentration by reducing the dissolution rate of berberin in gastric juice and also by prolonging the residence time of berberin in intestinal mucus. Nanocarriers have been used to localize berberine to the gastric epithelium for the treatment of *H. pylori* infection [10,11].

Chitosan (CS), a linear polysaccharide obtained by partial deacetylation of chitin, has been widely used in the biomedical field and drug delivery applications [12,13,14]. The naturally occurring polymer has many favorable characteristics, including mucoadhesive and pH-sensitive properties [15,16]. Chitosan-based nanoparticles have gained increasing attention for their efficient oral delivery of proteins and drugs [17,18]. Fucoidan (FD) is extracted from marine brown seaweed that has a backbone composed of sulfated esters of fucose and glucuronic acid or other monosaccharides [19]. Fucoidan can exert a wide variety of pharmacological activities, such as anti-inflammatory, anti-angiogenic, antitumor, and antithrombotic activities [20,21]. Suppression of inflammatory cytokine production in the Caco-2/RAW264.7 co-culture model by fucoidan was reported [22]. Moreover, recent studies have found that fucoidan enhanced epithelial barrier function via up-regulating the expression of the tight junction protein Claudin-1 [23].

Chitosan-based nanoparticles have been investigated in recent years for developing oral drug delivery carriers. However, the studies focused on preparing nanoparticles composed of a chitosan shell, thus the nanoparticles had the ablity to open the intestinal epithelial tight junctions. The nanoparticles were usually prepared by adding polyanions into excess amounts of chitosan solution to obtain nanoparticles covered with positively charged chitosan. In recent years, increased attention has been focused on the development of chitosan/fucoidan (CS/FD) complex nanoparticles for drug delivery [24,25,26,27,28,29,30]. Our previous study developed a chitosan/fucoidan (FD) nanoparticle with chitosan dominant at an outer layer. The highly positively charged nanoparticles could open the tight junction for the transport of anti-angiogenic sulfated polysaccharides across Caco-2 cell monolayers. However, the aim of this work was to develop a berberine-loaded chitosan/FD-Tau nanoparticles for treatment of the defective intestinal TJ barrier induced by bacterial endotoxin. Because berberine could attenuate pro-inflammatory cytokine-induced tight junction disruption, it should be targeted to the intestinal epithelial Caco-2 cells, but not the sublayer macrophage cells. Thus, the nanoparticles were not designed to open the tight junction for transepithelial transport of berberine.

To achieve the goal, FD was first conjugated with taurine (Tau) to obtain a fucoidan-taurine (FD-Tau) conjugate. Taurine can inhibit lipopolysaccharide-induced release of inflammatory factors to attenuate dysfunction in epithelial cells [31]. Moreover, the sulfonate group of taurine is a very strong acid which can increase the negative-charge density on fucoidan. Subsequently, a reverse of the CS/FD-Tau mixing process was developed to prepare negatively charged nanoparticles by adding CS solution into an excess amount of FD-Tau solution. This method was able to produce a FD-Tau-shelled nanoparticle because the excessive FD-Tau could be precipitated on the surface of the nanoparticles through the spontaneous formation of polyelectrolyte complex with chitosan. The nanoparticles shelled with FD-Tau are of benefit to the intestinal TJ barrier because fucoidan and taurine have been reported to attenuate dysfunction in epithelial cells. Furthermore, fucoidan can help the nanoparticles to target intestinal epithelial cells due to the fucose receptor on the epithelial cells. The prepared Ber-loaded CS/FD-Tau nanoparticles were then used for the treatment of defective intestinal epithelial TJ barrier caused by bacterial endotoxin. These nanoparticles were suitable to deliver berberine to epithelial Caco-2 cells without inducing the impairment of the intestinal barrier function. Berberine release from the nanoparticles could attenuate the intestinal epithelial impairment resulting from the inflammatory cytokine which was produced by endotoxin-activated macrophage. However, berberine could be delivered to the Caco-2 cell, thus the pro-inflammatory cytokines-mediated NF-κB signaling pathway in the cell could be inhibited and the epithelial TJ junction could be protected.

Moreover, the pH-sensitivity property and berberine release behavior of the Ber-loaded CS/FD-Tau nanoparticles were investigated in simulated gastric fluid (SGF) and intestinal fluid (SIF). The effect of the nanoparticles on LPS-induced TJ barrier dysfunction was investigated by measuring transepithelial electrical resistance (TEER) and paracellular flux of fluorescein isothiocyanate-dextran (FITC-dextran) in an intestinal epithelial Caco-2 cells/macrophage RAW264.7 cells co-culture system. Redistribution of ZO-1 TJ proteins was observed using confocal laser scanning microscopy (CLSM).

## 2. Results and Discussion

### 2.1. Characterization of FD-Tau Conjugate

**Figure 1:**
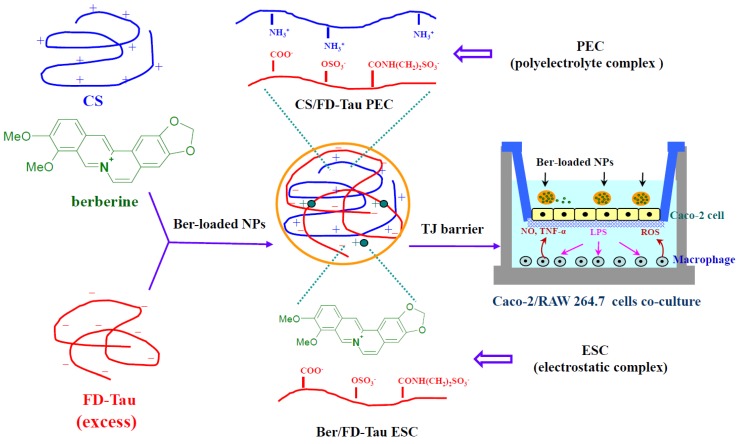
Schematic diagram of the preparation of berberine (Ber)-loaded chitosan (CS)/fucoidan-taurine (FD-Tau) nanoparticles and construction of the human colon carcinoma cell line (Caco-2 cells)/murine macrophage cell line (RAW 264.7 cells) co-culture system for estimating the protective effect of nanoparticles against lipopolysaccharides (LPS)-caused barrier dysfunction.

**Figure 2:**
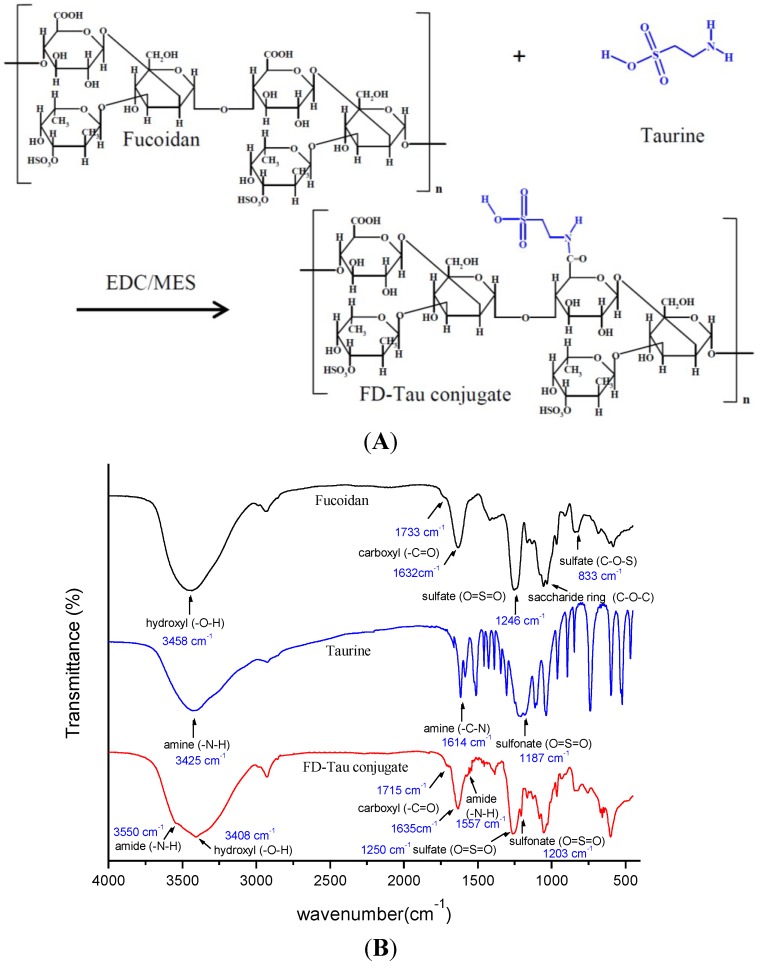
(**A**) Schematic diagram of the fucoidan-taurine (FD-Tau) conjugation reaction; (**B**) Fourier transform infrared (FTIR) spectra of fucoidan, taurine, and fucoidan-taurine (FD-Tau) conjugate.

A schematic diagram of the preparation of Ber-loaded CS/FD-Tau nanoparticles and construction of the Caco-2/RAW 264.7 cells co-culture system for estimating the protective effect of nanoparticles against LPS-caused barrier dysfunction is shown in Figure 1. Figure 2A shows the schematic diagram of the fucoidan-taurine (FD-Tau) conjugation reaction. FD-Tau conjugate was synthesized through activation of carboxyl groups by EDC in MES buffer followed by the formation of amide bonds between the activated carboxyl groups of fucoidan and the amino groups of taurine. As shown in Figure 2B, the FTIR spectrum of fucoidan (FD) shows the absorption band due to asymmetric and symmetric stretching vibration bands of O=S=O (1246 cm^−1^) and C–O–S stretch (833 cm^−1^), indicating the presence of a sulfate ester linkage. The carbonyl stretch C=O of a carboxylic acid appears at 1632 cm^−1^ and 1733 cm^−1^, indicating the presence of glucuronic acid in fucoidan. The FD-Tau conjugate demonstrated the asymmetric and symmetric stretching vibration bands of O=S=O at around 1203 cm^−1^ due to the sulfonate groups on taurine, while a lower intensity shoulder near 1557 cm^−1^ is due to the formation of amide bonds after conjugation. These results indicated that taurine has been successfully coupled to fucoidan. TNBS assay of the fucoidan/taurine reaction mixture showed 71.3% of unreacted primary amine of taurine. Accordingly, the taurine substitution ratio of the FD-Tau conjugate was estimated to be 223 μg taurine/mg FD-Tau conjugate.

### 2.2. Characterization of Berberine-Loaded Nanoparticles

Fucoidan is a polysaccharide extracted from marine brown seaweed which contains negatively charged sulfate and carboxyl groups. The electrostatic interactions between fucoidan and oppositely charged chitosan lead to the formation of stable colloidal nanoparticles. However, the nanoparticles were found to disintegrate in simulated gastric fluid (SGF, pH 2.0) rapidly because fucoidan contains carboxyl groups which are less acidic (pKa near 3.0). In this study, a FD-Tau conjugate was pre-synthesized to reduce the carboxyl groups and increase the more acidic sulfonate groups (pKa 1.5). Berberine is an isoquinoline alkaloid possessing a strong positive charge on its quaternary ammonium group. The FD-Tau conjugate was able to assemble with berberine to form stable Ber/FD-Tau nanoparticles. The particle size of Ber/FD-Tau nanoparticles could be varied over a wide range by adjusting the weight ratio of berberine to FD-Tau. As shown in Table 1, the average particle size increased from 111.7 ± 2.7 to 156.3 ± 5.5 nm by decreasing the Ber/FD-Tau weight ratio from 2/1 to 2/4. The zeta potentials were negative and also decreased with the decrease of the Ber/FD-Tau weight ratio, demonstrating that FD-Tau was increasingly exposed on the surfaces of the nanoparticles. The large polydispersity (PDI) of the Ber/FD-Tau nanoparticle systems indicated a wide range of particle size distribution. Moreover, berberine-loading efficiency was low (Table 1), suggesting that berberine was poorly retained in the nanoparticles because berberine and FD-Tau could not produce well-organized nanoparticles.

**Table 1:** Average particle size, particle size distribution (polydispersity, PDI), zeta potential, berberine-loading content of Ber/FD-Tau nanoparticles prepared from berberine (Ber) (1.0 mg/mL, 1 mL) and fucoidan-taurine (FD-Tau) (0.5, 1.0, 1.5, 2.0 mg/mL, 1 mL) aqueous solution.

FD-Tau (mg/mL)	Ber/FD-Tau Weight Ratio	Average Size (nm)	Zeta Potential (mV)	Drug Loading (%)	PDI
0.5	2/1	111.7 ± 2.7	–14.7 ± 1.4	10.3 ± 0.4	0.41 ± 0.01
1.0	2/2	120.9 ± 4.1	–20.4 ± 0.7	13.1 ± 0.5	0.39 ± 0.02
1.5	2/3	147.4 ± 4.8	–27.1 ± 0.9	9.7 ± 0.8	0.47 ± 0.01
2.0	2/4	156.3 ± 5.5	–31.4 ± 1.8	12.8 ± 0.3	0.46 ± 0.02

**Figure 3:**
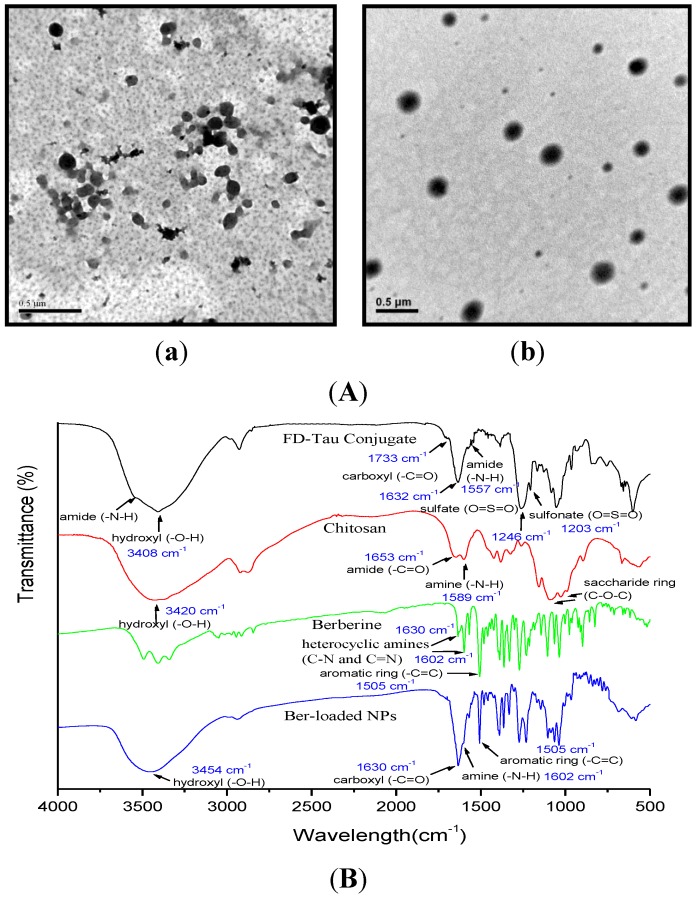
(**A**) Transmission electron microscopy (TEM) micrographs of Ber/FD-Tau (**a**) and Ber-loaded CS/FD-Tau (**b**) nanoparticles; (**B**) FTIR spectra of FD-Tau conjugate, chitosan, berberine, and Ber-loaded nanoparticles.

To enhance the berberine-loading efficiency of the nanoparticle system, chitosan was employed to form a polyelectrolyte complex with FD-Tau to obtain Ber-loaded CS/FD-Tau nanoparticles. We found that stable nanoparticles could only be prepared if both concentrations of berberine and FD-Tau were no more than 1.0 mg/mL. Therefore, the concentrations of berberine and FD-Tau were kept at 1.0 mg/mL in order to form a polyelectrolyte complex with chitosan. As shown in Table 2, the zeta potentials of the CS/FD-Tau nanoparticles were almost negative, suggesting that the reversed mixing process used in this study was able to produce FD-Tau-shelled nanoparticles because the excessive FD-Tau could be precipitated on the surface of the nanoparticles. The average particle size of Ber-loaded CS/FD-Tau nanoparticles increased noticeably when the chitosan amount was increased. In the presence of positively charged berberine, low-positively charged nanoparticles could be produced, depending on the chitosan/berberine/FD-Tau weight ratios (Table 2). Figure 3A shows the TEM micrographs of Ber/FD-Tau and Ber-loaded CS/FD-Tau nanoparticles. The Ber/FD-Tau nanoparticles are irregular in shape while the Ber-loaded CS/FD-Tau nanoparticles are spherical. Figure 3B shows the FTIR spectra of berberine, FD-Tau, chitosan and the Ber-loaded nanoparticles. The charcteristic bands of berberine at 1602 cm^−1^ (quaternary iminium ion, -C=N-) and 1505 cm^−1^ (C=C stretching vibration in the aromatic ring) are observed from the spectrum of the Ber-loaded nanoparticles. The characteristic peak of chitosan at 1589 cm^−1^ (the amino group, -NH_2_) and the absorption peak of fucoidan at 1632 cm^−1^ (carboxylic ion, -COO^−^) respectively shift to 1602 and 1630 cm^−1^, accompanied by alteration in intensity of band signals. These results suggest that berberine was successfully incorporated in the nanoparticles through the formation of polyelectrolyte complex between fucoidan and chitosan.

**Table 2:** Average particle size, particle size distribution (polydispersity, PDI), zeta potential, berberine-loading content of Ber-loaded CS/FD-Tau nanoparticles (NPs) prepared from the mixed solutions (1 mL) of CS/Ber with different weight ratios and FD-Tau aqueous solution (1.0 mg/mL, 2 mL).

CS/Ber (mg/mg)	CS/Ber/FD-Tau Weight Ratio	Average Particle Size (nm)	PDI	Zeta Potential (mV)	Ber-Loading Content (%)
CS/FD-Tau NPs					
0.5/0.0	1/0/4	225.6 ± 3.4	0.32 ± 0.02	−38.2 ± 1.4	−
1.0/0.0	2/0/4	208.1 ± 5.5	0.35 ± 0.02	−35.7 ± 2.1	−
1.5/0.0	3/0/4	204.5 ± 4.2	0.29 ± 0.02	−11.7 ± 1.7	−
2.0/0.0	4/0/4	179.7 ± 3.9	0.33 ± 0.02	+17.1 ± 1.6	−
Ber-loaded CS/FD-Tau NPs					
0.5/1.0	1/2/4	145.9 ± 2.7	0.27 ± 0.01	−13.1 ± 0.8	32.3 ± 0.4
1.0/1.0	2/2/4	187.4 ± 6.2	0.21 ± 0.01	+7.6 ± 0.5	50.1 ± 2.5
1.5/1.0	3/2/4	359.4 ± 4.7	0.39 ± 0.02	+15.6 ± 0.6	62.7 ± 3.4
2.0/1.0	4/2/4 *	−	−	−	−

*: Aggregation and precipitation of nanoparticles were found at CS/Ber/FD-Tau weight ratio of 4/2/4.

### 2.3. Berberine Release

**Figure 4:**
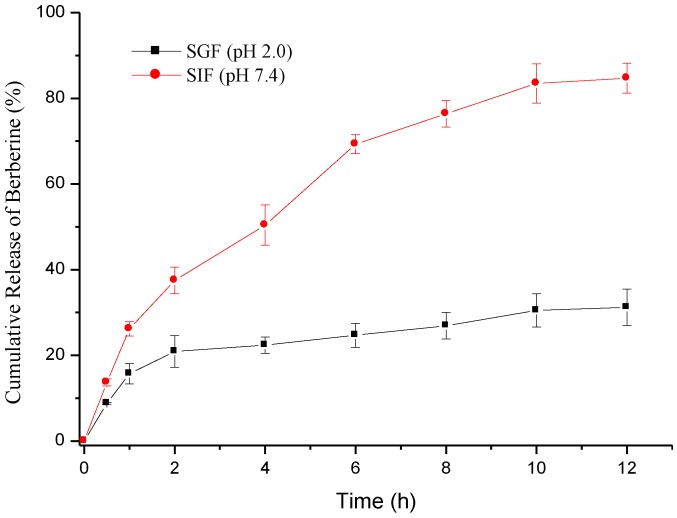
Cumulative release of berberine from nanoparticles at pH 2.0 (simulated gastric fluid, SGF) and pH 7.4 (simulated intestinal fluid, SIF).

The loading efficiency and PDI of the Ber-loaded CS/FD-Tau nanoparticles prepared at a CS/Ber/FD-Tau weight ratio = 2/2/4 were 50.1% ± 2.5% and 0.21 ± 0.01, respectively (Table 2). Therefore, the nanoaprticles with optimal drug loading and size distribution were chosen for drug release and cell culture. The sustained release profile of berberine from nanoparticles was investigated in the dissolution mediums of SGF (pH 2.0) and SIF (pH 7.4). Factors affecting the berberine release rate were the hydrophilicity and stability of various nanoparticle systems and the pH of the release medium. Fucoidan is a polysaccharide composed of sulfated esters of fucose and glucuronic acid. The pKa values of sulfate esters in sulfated fucose are around 1.5 while the pKa value of the carboxylate group in glucuronic acid is about 3.0. At pH 2.0, strong acid protonated the carboxylate ions (-COO^−^) in fucoidan, the polyelectrolyte complex of chitosan and fucoidan thus could be rapidly broken down [28]. After chemical modification, the carboxylate group decreased in amount but the sulfonate group increased due to the conjugation of taurine with the glucuronic acid residue of fucoidan. Accordingly, more negative charge was retained on the FD-Tau conjugate in SGF than on the original fucoidan. The burst release was not obvious because the positively charged berberine could form a strong electrostatic interaction with FD-Tau during the preparation of the nanoparticles. As shown in Figure 4, after 12 h of dissolution test at pH 2.0, there was only 31.2% berberine released from the Ber-loaded nanoparticles. Under these conditions, the protonaed chitosan (pKa value of the primary amine is around 6.5) and negatively charged FD-Tau conjugate can form stable and well-organized nanoparticles, thus providing a slow and continuous release of berberine. On the other hand, at neutral pH (pH 7.4), chitosan was deprotonated and berberine was rapidly released from the disintegrating nanoparticles [10]. It is worth noting that, at pH 7.4, the electrostatic attractions between chitosan and FD-Tau conjugate became weaker than those at pH 2.0 because chitosan was deprotonated. The nanoparticles released 85.7% of loaded berberine within 12 h (Figure 4). The result suggests that the CS/Tau-FD nanoparticles can release berberine in response to the change in pH value of the simulated gastrointestinal fluids. The intestinal pH (near neutrality) facilitates the release of berberine, indicating that the nanoparticles may be a potential intestinal delivery carrier for berberine.

### 2.4. Cytotoxicity of Berberine and Nanoparticles

**Figure 5:**
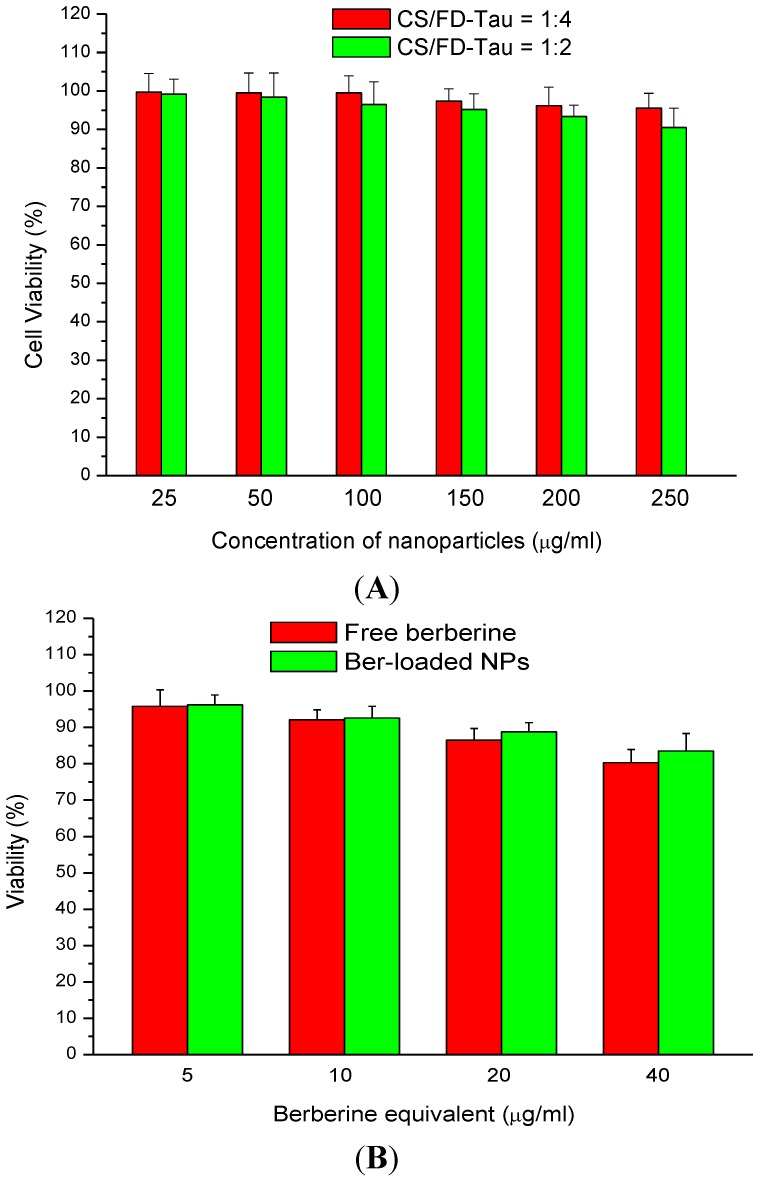
Dose-dependent cytotoxicity of berberine-free nanoparticles (**A**) and berberine-loaded nanoparticles (**B**) on Caco-2 cells.

The berberine-free nanoparticles did not exhibit significant cytotoxicity. Cell viability of Caco-2 cells was higher than 90% by treating the cells with 250 μg/mL nanoparticles (Figure 5A). Figure 5 shows the dose-dependent cytotoxicity of Ber-loaded nanoparticles on Caco-2 cells. It was reported that cell viability of A549 cells incubated with CS/FD nanoparticles at a concentration below 3 mg/mL was higher than 80% [29]. Both berberine and Ber-loaded nanoparticles induced low cytotoxicity in the concentration range of 5–40 μg/mL as measured by the MTT assay (Figure 5B). Therefore, the nanoparticle samples were used at a Ber equivalent concentration of 30 μg/mL for the following studies.

### 2.5. Inhibition of NO and TNF-α Production

Bacterial-derived lipopolysaccharides (LPS) can cause impaired barrier function, leading to a leak-flux diarrhea by enhancing uptake of antigen across the intestinal epithelium tight junction (TJ). Most types of intestinal inflammation are associated with disorder of the intestinal epithelial TJ barrier function. LPS regulates proinflammatory cytokine which is produced by macrophage and can cause intestinal epithelial barrier dysfunction. The protective effect of Ber-loaded nanoparticles on epithelial barrier function of Caco-2 cell monolayer co-cultured with LPS-treated RAW 264.7 cells was evaluated by TEER and FITC-dextran permeability.

**Figure 6:**
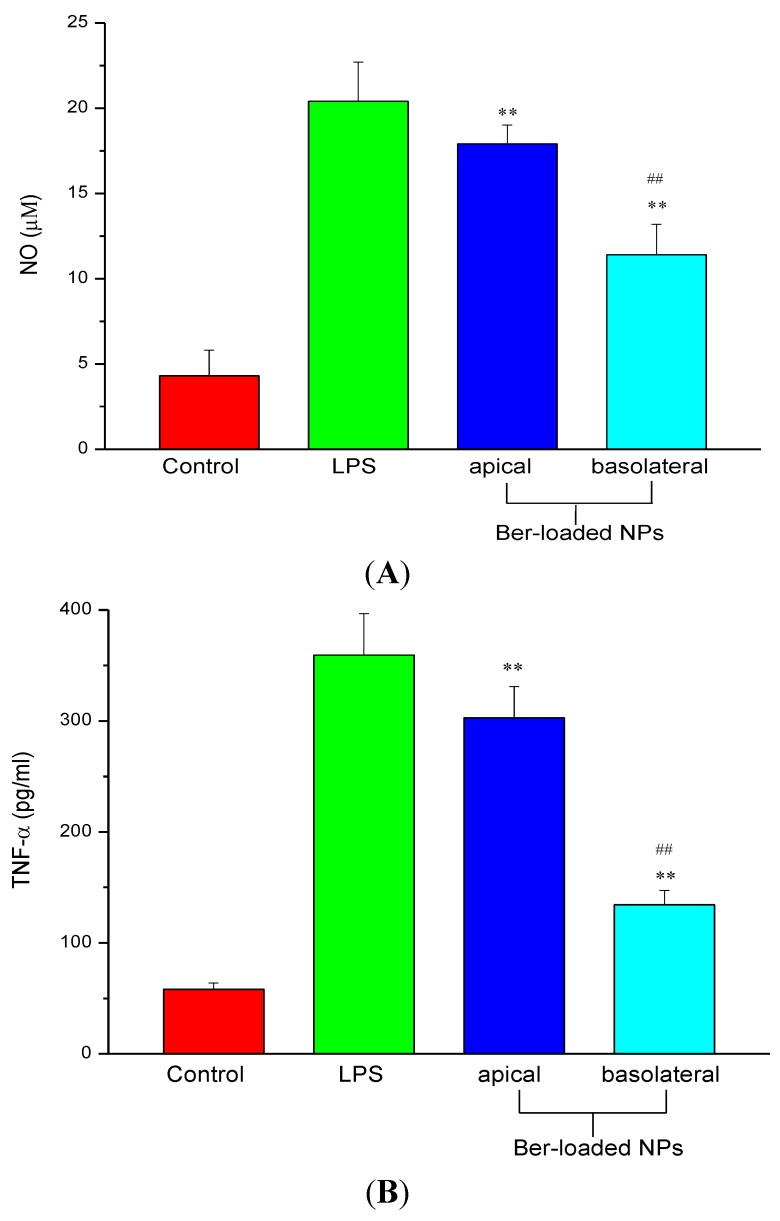
(**A**) Nitric oxide (NO) and (**B**) TNF-α production in RAW264.7 cells stimulated by 100 ng/mL of LPS in presence of berberine-free nanoparticles (NPs) and berberine-loaded nanoparticles (Ber-loaded NPs) by adding 30 μg/mL berberine equivalent of the nanoparticles to the apical and basolateral sides respectively (*n* = 5). ** *p* < 0.01 compared with the control; ## *p* < 0.01 compared with the LPS-treated group.

To identify the protective effect of the Ber-loaded nanoparticles on the tight junction which was disrupted by inflammation, macrophages were incubated with the nanoparticles alone or in the presence of LPS. LPS is known to secrete inflammatory cytokines like TNF-α which are involved in immune responses and induces epithelial destruction [32]. LPS-stimulated nitric oxide (NO) production in macrophage can be easily determined and used as an index of inflammation. As shown in Figure 6, without LPS, the presence of Ber-free nanoparticles (control) produced only low amounts of NO and TNF-α protein. Yet, the presence of PLS alone induced a large amount of them. As evidenced from Figure 6A, further addition of Ber-loaded nanoparticles to the basolateral side considerably inhibited NO production in LPS-activated macrophages. The reduction in NO production indicated that iNOS protein expression, a key factor in oxidant-induced disruption of intestinal Caco-2 monolayer barrier, was inhibited by the Ber-loaded nanoparticles. Moreover, the expression of TNF-α protein by LPS-activated macrophages was markedly inhibited by the Ber-loaded nanoparticles (Figure 6B). This agrees with the report from Jeong *et al*., that berberine could inhibit LPS-induced expression of proinflammatory genes in peritoneal macrophages and RAW 264.7 cells [33]. Besides, chitosan-based nanoparticles have been widely studied as drug carriers in mucosal and targeting drug delivery [34,35].

However, the addition of Ber-loaded nanoparticle to the apical side did not significantly reduce NO and TNF-α production by RAW 264.7 cells. The results suggest that the nanoparticles cannot effectively permeate through the TJ barrier to inhibit the inflammatory response in macrophage. The nanoparticles must be localized to macrophage and subsequently release berberine to inhibit the production of NO and pro-inflammatory cytokines by macrophage.

### 2.6. Intestinal TJ Permeability

**Figure 7:**
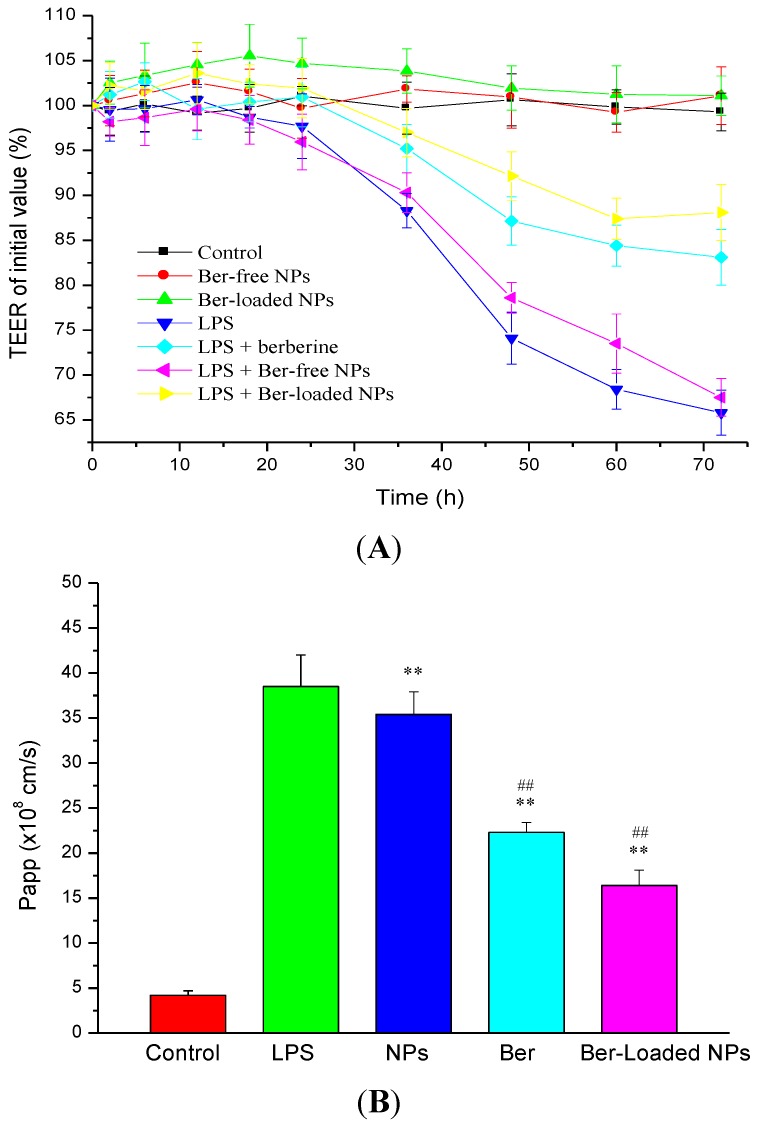
Epithelial barrier function determined by measurements of transepithelial electrical resistance (TEER) (**A**) and Fluorescein isothiocyanate (FITC) labeled dextrans (FITC-dextran) permeability (**B**). Caco-2 cell monolayers were co-cultured with RAW264.7 stimulated by 100 ng/mL of LPS in the presence of berberine (Ber), and berberine-free (NPs) and loaded nanoparticles (Ber-loaded NPs, 30 μg/mL berberine equivalent) (*n* = 5). ** *p* < 0.01 compared with the control; ## *p* < 0.01 compared with the LPS-treated group.

As shown in Figure 7A, Ber-free nanoparticles or Ber-loaded nanoparticles alone did not result in reduction in TEER. The zeta potentials of the nanoparticles were −35.7 ± 2.1 and 7.6 ± 0.5 mV, respectively (Table 2). Berberine and fucoidan have been shown to improve intestinal barrier function and reduce intestinal epithelial permeability [6,23]. On the other hand, when the LPS (100 ng/mL) was applied to the basolateral compartment, it caused a significant decrease in TEER after 48 h incubation with the co-culture system of intestinal epithelial Caco-2 cells/macrophage RAW264.7 cells. However, the decrease in TEER induced by LPS-activated macrophage could be retarded by berberine treatment. To determine whether the Ber-loaded nanoparticles could reduce inflammation-induced TJ disruption, polarized Caco-2 monolayer was exposed to the nanoparticles after LPS treatment; and the TEER was monitored for 72 h. We found that the released berberine could effectively inhibit the LPS-induced disruption of TJ. For comparison, by adding Ber-free nanoparticles after LPS treatment, the decreasing trend of the TEER value was relatively close to that of the negative control group (LPS treatment). This indicated that the nanoparticles did not exhibit a significant protective effect on epithelial cell injury. Moreover, it was found that TEER values of Caco-2 monolayer exposed to the Ber-loaded nanoparticles 2 h before LPS treatment remained at a high level. These results suggested that the preventive effect of nanoparticles on LPS-induced destruction of the intestinal epithelial barrier was dependent on the incorporated berberine.

LPS play an essential role in inducing inflammatory response in macrophages and the subsequent TJ disassembly in intestinal epithelia accompanied by an increase in paracellular permeability. The effect of LPS-induced intestinal epithelial barrier dysfunction was determined by measuring the paracellular flux of FITC-dextran. The permeability of TJ increased after stimulating the cell monolayer with LPS. As shown in Figure 7B, LPS caused a significant increase in the permeability coefficient (Papp) of FITC-dextran. The increase in FITC-dextran flux from the apical to the basal side corresponded to the increase in the Caco-2 TJ permeability as indicated by the increased TEER. However, as the Caco-2 intestinal monolayer was co-treated with the LPS and Ber-loaded nanoparticles, the permeability of FITC-dextran through the Caco-2 cells was significantly reduced. The Ber-loaded nanoparticles thus lessened the increase of paracellular flux induced by LPS. These results indicated that Ber-loaded nanoparticles can be used to reduce the effects of LPS-induced impairment of epithelial barrier function.

Although the above mentioned results suggested that the Ber-loaded nanoparticle cannot effectively permeate through TJ barrier to inhibit the production of NO and pro-inflammatory cytokines by macrophage (Figure 6A,B), the effect of the nanoparticles on attenuating inflammation-induced tight junction dysfunction was significant (Figure 7A,B). Berberine is a traditional Chinese medicine used for the treatment of gastroenteritis and infective diarrhea. Berberine could be delivered to the Caco-2 cell, but not to the sublayer macrophage, thus the pro-inflammatory cytokines mediating NF-κB signaling pathway in the cell could be inhibited to protect the TJ junction. In this study, the decrease in TEER and increase in paracellular permeability induced by LPS were moderated by Ber-loaded nanoparticles. Berberine has a considerable effect in decreasing intestinal epithelial permeability without affecting TJ proteins. Li *et al*., (2010) reported that berberine can inhibit pro-inflammatory cytokines such as TNF-α and IFN-γ induced intestinal epithelial tight junction damage [8]. Another study showed that increased intestinal permeability and tight junction disruption induced by LPS in mice could be efficaciously suppressed by berberine [6]. Those results revealed that berberine plays an important role in ameliorating intestinal epithelial TJ damage. On the other hand, fucoidan demonstrated superior immunomodulatory activity of suppressing TNF-α production in macrophage. Fucoidan also improves intestinal epithelial barrier function by reducing IL-8 gene expression and upregulating the expression of claudin-1 in Caco-2 cells [36,37]. Moreover, taurine is a potent compound against intestinal inflammation by repressing TNF-α induced Caco-2 cell damage [38]. Incorporation of berberine with the FD-Tau conjugate into the nanoparticle system was thus able to increasingly protect the intestinal epithelial barrier function under LPS-induced inflammation by macrophage, compared with berberine alone in solution (Figure 7A,B). Moreover, the Ber-loaded nanoparticles anchored with the mucoadhesive polymer, chitosan, might prolong the residence time of the nanoparticles on the intestinal mucous layer and subsequently localize the released berberine to the intestinal epithelial cells in an inflammatory bowel disease.

### 2.7. Immunostaining of ZO-1 Protein

**Figure 8:**
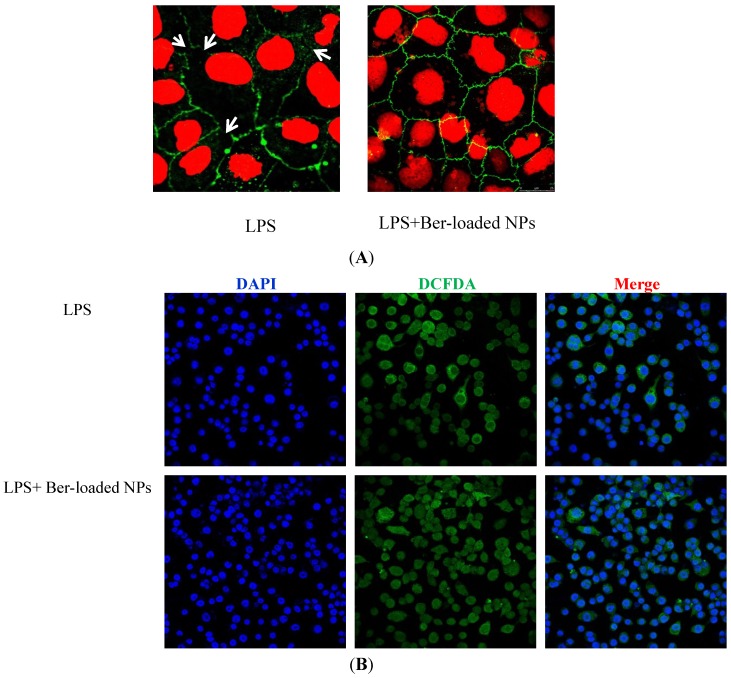
(**A**) Confocal laser scanning microscopy (CLSM) visualization of protective effect of berberine in TNF-α induced epithelial barrier disruption. ZO-1 protein of Caco-2 cells treated without (**a**) or with (**b**) berberine-loaded nanoparticles (Ber-loaded NPs) in the absence of TNF-α for 72 h; (**B**) CLSM visualization of intracellular ROS levels in RAW 264.7 cells treated without (**a**) or with (**b**) berberine-loaded nanoparticles in the absence of LPS for 72 h.

LPS is known to induce intestinal barrier dysfunction and disruption of epithelial tight junction due to the production of pro-inflammatory cytokines such as TNF-α. The effect of TNF-α on the epithelial barrier function is associated with the redistribution of TJ proteins in polarized Caco-2 cell. Zonula occludens-1 (ZO-1) is a tight junction protein that links transmembrane proteins of the TJ to the actin cytoskeleton. As shown in Figure 8A, in the control group (without TNF-α), the stained Caco-2 cell visualized by CLSM exhibited a normally smooth ZO-1 distribution. Exposure of Caco-2 cells to TNF-α led to slight disruption of the TJ structure in polarized Caco-2 monolayers. The disrupted ZO-1 proteins were not recovered after the removal of TNF-α, suggesting that the TJ was irreversibly disrupted by LPS. Treatment of the cells by the Ber-loaded nanoparticles prevented the TJ protein from disruption by TNF-α. The ZO-1 proteins remained intact after directly treating Caco-2 cells with TNF-α in the presence of the nanoparticles (Figure 8A). Several studies have demonstrated that berberine or fucoidan could directly induce changes in TJ protein expression, consequently improving intestinal epithelial barrier functions.

ROS is known to induce disruption of intestinal epithelial barrier function [39,40]. CLSM was carried out to investigate the oxidative stress induced by LPS in RAW 264.7 cells and the ROS scavenging activity of the nanoparticles in the LPS-treated cells. The fluorescent images of intracellular ROS levels in RAW 264.7 cells after 24 h of incubation with LPS are shown in Figure 8B. The green fluorescence was from ROS-sensitive fluorescence dye (DCFDA) whereas the blue fluorescence was from DAPI dye. The merged image of the fluorescence from DCFDA and DAPI dyes showed that the blue fluorescence in nuclei was surrounded by green fluorescence. Visualization of the green fluorescent signal in the cells indicated the production of intracellular oxidative stress by LPS. Pretreatment of the cells with 30 μg/mL berberine equivalent of nanoparticles quenched the green fluorescence of the ROS-sensitive dye in RAW 264.7 cells (Figure 8B). The nanoparticles therefore seemed to be a promising carrier to deliver berberine to scavenge the intracellular ROS in macrophage.

## 3. Experimental Section

### 3.1. Materials

Lipopolysaccharides (LPS) from *Salmonella enterica* serotype typhimurium (L6143), Taurine, 1-ethyl-3-(3-(dimethylamino)propyl)carbodiimide hydrochloride (EDC), berberine, Dulbecco’s modified Eagle medium (DMEM), 2-(*N*-Morpholino)ethanesulfonic acid sodium salt (MES), Griess reagent, berberine, fluorescein isothiocyanate–dextran (FITC-dextran, 4000 kD), 2′,7′-Dichlorofluorescin diacetate (DCFDA), and MTT reagent were purchased from Sigma-Aldrich (St Louis, MO, USA).

### 3.2. Synthesis and Characterization of Fucoidan-Taurine (FD-Tau) Conjugates

FD-Tau conjugates were synthesized following a modified procedure described by Yu *et al*. [41]. Briefly, the carboxyl group on the glucuronic acid residues of fucoidan (MW 80 kDa, NOVA Pharma & Liposome Biotech Co., Ltd, Kaohsiung, Taiwan) was activated by EDC in MES buffer (25 mM, pH 5.0) and the activated fucoidan solution was added with an adequate amount of taurine (weight ratio of fucoidan/taurine/EDC = 1:1:1.5), and the resulting mixture was allowed to react for 48 h. Both reactions were quenched by adding hydroxylamine, and adjusting the pH of the reaction medium to 8.0. The obtained FD-Tau polymer conjugates were dialyzed against water and then lyophilized. The purified products were analyzed by FTIR spectrometer (Perkin-Elmer Spectrum RX1, Waltham, MA, USA). The taurine substitution ratio of the FD-Tau conjugate was estimated by reacting free taurine with 2,4,6-trinitrobenzenesulfonic acid (TNBS) and subsequently measuring the absorbance at 420 nm.

### 3.3. Preparation of Berberine-Loaded CS/FD-Tau Nanoparticles

Berberine and FD-Tau solutions were prepared by respectively dissolving adequate amounts of berberine and FD-Tau in DI water. Berberine/FD-Tau nanoparticles were then prepared by adding the berberine solution (1.0 mg/mL, 1 mL) into the FD-Tau solution (0.5, 1.0, 1.5, 2.0 mg/mL, 1 mL) at various weight ratios (as shown in Table 1). Berberine-loaded CS/FD-Tau nanoparticles were prepared by dissolving chitosan (MW 60 kDa, deacetylation degree 85%, Koyo Chemical Co. Ltd. Hyogo, Japan) and berberine (1.0 mg/mL) together in DI water at different chitosan-to-berberine weight ratios (as shown in Table 2). Subsequently, the berberine/chitosan solution (chitosan/berberine weight ratio = 0.5 mg/1.0 mg, 1.0 mg/1.0 mg, 1.5 mg/1.0 mg, and 2.0 mg/1.0 mg, 1 mL) was added to the aqueous FD-Tau solution (1.0 mg/mL, 2 mL) to obtain self-assembled nanoparticles. A Malvern Zetasizer (3000HS, Worcestershire, UK) was used to measure the particle size and zeta potential of the nanoparticle suspensions. Transmission electron microscopy (TEM, Hitachi H-600, Tokyo, Japan) was used observe the morphology of nanoparticles after drying the suspension on a copper grid (300 mesh) coated with carbon film.

### 3.4. In Vitro Release Study

The nanoparticles were ultracentrifugated to remove free berberine molecules; and the loading efficiency (LE) was determined by measuring the concentration of free berberine in the supernatant using high performance liquid chromatography (HPLC) coupled with an ultraviolet (UV) absorption detector operated at 360 nm. Quantification of berberine was performed using a reverse-phase C18 column (150 mm × 4.6 mm, 5 μm), with a mobile phase of acetonitrile—0.04 M H_3_PO_4_ (42:58 vol.%)—and a flow rate of 1.0 mL/min. Berberine release from the nanoparticles was investigated in SGF (0.01 N HCl, pH 2.0) and SIF (PBS, pH 7.4) at 37 °C under agitation. Samples were collected at several pre-determined times. After centrifugation, the supernatants were used for berberine assay. The fraction of drug release was calculated based on the initial amount of berberine incorporated in the nanoparticles. The release study was repeated four times for each time point to obtain the average and standard deviation (SD).

### 3.5. MTT Assay for Cell Viability

The Caco-2 cell (BCRC 60182, Hsinchu, Taiwan) was maintained in DMEM medium containing 10% fetal bovine serum (FBS), 1% penicillin (100 U/mL)-streptomycin (100 μg/mL), and 4mM glutamine. Caco-2 cells were cultured in 96-well plates at a density of 2 × 10^4^ cells per well. Subsequent to adherence, berberine (5, 10, 20 and 40 μg/mL), Ber-loaded nanoparticles (the same berberine equivalents), Ber-free nanoparticles (25, 50, 100, 150, 200 and 250 μg/mL) were added to the cells and incubated for 24 h. After 48 h incubation at 37 °C, the medium was aspirated and the cells were washed with PBS. The colorimetric determination of cell viability was performed by adding 20 μL MTT solution (5 mg/mL) to each well. After 4 h of incubation, the culture medium was removed carefully. The supernatant was aspirated, and 100 μL dimethyl sulfoxide (DMSO) was added to dissolve the formazan crystal. The optical intensity of color (absorbance) was measured on a Perkin Elmer EnSpire 2300 multimode plate reader (PerkinElmer, Inc., Waltham, MA, USA) at 570 nm. Cytotoxicity was expressed as the relative viability (% control).

### 3.6. Caco-2/Macrophage Co-Culture System

Caco-2 cells were seeded at 4 × 10^5^ cells/well and monolayers were grown in Costar Transwell 6 wells/plates (Corning Costar Corp., NY, USA) with a 3 μm pore size filter insert. The electrical resistances of the filter-grown monolayers were measured using a Millicell^®^-Electrical Resistance System (Millipore Corporation, Billerica, MA, USA). Monolayers were maintained for 14–20 days in an atmosphere of 95% air and 5% CO_2_ at 37 °C until the transepithelial electrical resistance (TEER) reached a value in the range of 900–1000 Ω cm^2^. RAW264.7 cells were seeded into the bottom plate at a density of 7.5 × 10^5^ cells/well and the insert with polarized Caco-2 monolayer was added into the Transwell plate preloaded with RAW264.7 cells (BCRC 60001, Hsinchu, Taiwan). The anti-inflammatory effect of Ber-loaded nanoparticles was examined by addition of 100 ng/mL LPS to the basolateral side subsequent to adding 30 μg/mL berberine equivalent of the nanoparticles to the apical side. For comparison, 30 μg/mL berberine equivalent of the nanoparticles was directly added to the basolateral side (RAW264.7 cells). Nitric oxide and TNF-α productions were determined by culture supernatant collected from the basolateral side after 24 h.

### 3.7. Colorimetric Nitric Oxide Assay

Griess reagent (0.1% *N*-(1-naphthyl)-ethylenediamine dihydrochloride and 1% sulfanilamide) was used to measure LPS-induced nitric oxide levels (NO) in RAW 264.7 macophage. At the end of incubation, 50 μL of Griess solution was added to the same volume of the supernatant for 10 min. The absorbance of the final product was measured at a wavelength of 540 nm using a Perkin Elmer EnSpire 2300 multimode plate reader to determine the nitrite concentration with a calibration curve constructed using a standard solution of NaNO_2_.

### 3.8. Enzyme-Linked Immunosorbent Assay (ELISA) for TNF-α

Subsequent to LPS treatment, the TNF-α concentration in culture supernatant was determined by a sandwich enzyme-linked immunosorbent assay (ELISA). Thermo Scientific 96-well immunoplate was coated with anti-mouse TNF-α monoclonal antibody (R & D Systems) and blocked using 4% bovine serum albumin (BSA) in PBS. After the addition of 100 μL diluted sample to the wells, an anti-mouse TNF-α polyclonal antibody (R & D Systems, Minneapolis, MN, USA) was added to the ELISA plate. The plate was developed by horseradish peroxidase for 2 h and the color development was terminated with the addition of a 1 N H2SO4 solution. The absorbance was read at 450 nm using a Perkin Elmer EnSpire 2300 multimode plate reader (PerkinElmer, Inc., Waltham, MA, USA).

### 3.9. Measurement of TEER and Paracellular Permeability

*S. typhimurium* LPS (100 ng/mL, L6143, Sigma Chemical Co., St. Louis, MO, USA) was added to the basolateral side (cultured with RAW264.7 cells) of Transwell plate to induce the inflammatory response. The effect of the Ber-loaded nanoparticles on attenuating LPS-induced tight junction disruption was evaluated by adding 30 μg/mL berberine equivalent of the nanoparticles to the apical side (insert with Caco-2 cells monolayer). For comparison, the system in which the cells were not treated by LPS was used as a control. Moreover, the cells were pre-treated with 30 μg/mL berberine equivalent of the nanoparticles for 2 h, followed by adding LPS to RAW264.7 cells to induce tight junction damage in Caco-2 monolayer. Changes in TEER were measured using the above mentioned method.

The transepithelial permeability was quantified by measuring the paracellular flux of fluorescein isothiocyanate (FITC) labeled dextrans (FITC-dextran). Transport of FITC-dextran (500 μg/mL in the apical compartment) across Caco-2 cell monolayer was quantitatively analyzed by measuring fluorescence intensity in the receiver compartment (basolateral side) at different time periods. The intensity of fluorescence emission (FL intensity) was determined by a Perkin Elmer EnSpire 2300 multimode plate reader, with excitation and emission wavelengths set to 488 and 519 nm, respectively. The amount of transported FITC-dextran was calculated using the calibration curve of FITC-dextran. The apparent permeability coefficient (Papp) was determined as follows:

Papp (cm/s) = (Δ*Q/*Δt)/(A × *C_0_*)

where Δ*Q/*Δt (μg/s) is the cumulative amount transported, *A* is the diffusion area (4.67 cm^2^), and *C_0_* is the initial FITC-dextran concentration in the donor side (μg/mL).

### 3.10. CLSM Visualization of Immunostained TJ Protein

Cells grown on glass cover slips in a 6-well plate were directly incubated with the pro-inflammatory cytokines, TNF-α (10 ng/mL), in the presence or absence of the Ber-loaded nanoparticles (30 μg berberine equivalent/mL). After removal of culture medium, cells were fixed with 3.7% paraformaldehyde. Subsequently, cells were permeabilized with 0.1% Triton X-100 at room temperature for 10 min. The cells were incubated with rabbit anti-ZO-1 monoclonal antibody (Zymed Laboraties, Inc., San Francisco, CA, USA) and subsequently stained by a Cy-3 conjugated goat anti-rabbit IgG (Jackson ImmunoResearch Laboratories, West Grove, PA, USA). Dislocation of ZO-1 protein in Caco-2 cells was examined under a confocal laser scanning microscopy (CLSM, Leica TCS SP2, Bensheim, Germany). To determine reactive oxygen species (ROS) induced by LPS (100 ng/mL) in RAW264.7 cells, the culture medium was removed and the cells were washed with PBS. Subsequently, the cells were incubated in the dark with DCFDA (20 μM) which could passively diffuse across the cell membrane. The diacetate of nonfluorescent DCFDA was cleaved by intracellular esterase and then reacted with intracellular ROS to produce fluorescence. Fluorescence images of ROS generated in RAW264.7 cells were visualized using CLSM.

### 3.11. Statistical Analysis

All measurements were replicated three times and data were expressed as the mean ± standard deviation. Statistical analysis was performed by one-way analysis of variance and the determination of confidence intervals at *p* < 0.05 using SAS version 9.1 (SAS Institute, Cary, NC, USA).

## 4. Conclusions

In this work, Ber-loaded CS/FD-Tau nanoparticles were developed and acted as an epithelial protective material to prevent redistribution of TJ protein caused by bacterial endotoxin (LPS). The pH-responsive nanoparticles were stable at pH 2.0 but became unstable as the pH increased to 7.4. The release rate of berberine from the nanoparticle was slow in SGF but fast in SIF. Measurements of TEER and paracellular flux of FITC-dextran in Caco-2 intestinal monolayers showed that the Ber-loaded nanoparticles were able to diminish the LPS-induced increment in intestinal epithelial TJ permeability. CLSM confirmed that the Ber-loaded nanoparticles prevented redistribution of ZO-1 proteins mediated by TNF-α induced TJ disruption. These findings suggested that the Ber-loaded nanoparticle is a potential carrier for site-specific delivery of berberine to the intestine for the inhibition of impaired intestinal barrier function. The nanoparticles might serve as an appropriate therapy for the treatment of disease associated with intestinal epithelial TJ dysfunction.
